# The benefits, challenges and impacts of accessing social media group support for breastfeeding: A systematic review

**DOI:** 10.1111/mcn.13399

**Published:** 2022-07-12

**Authors:** Holly Morse, Amy Brown

**Affiliations:** ^1^ Department of Public Health, Policy and Social Sciences Swansea University Swansea UK; ^2^ Centre for Lactation, Infant Feeding and Translation Research (LIFT) Swansea University Swansea UK

**Keywords:** breastfeeding, lactation support, mothers, online social support, psychosocial support, social media

## Abstract

Breastfeeding support is a key component in meeting the public health responsibility of increasing breastfeeding rates, with access to individualised, convenient and linked support across services central to improved outcomes. With the rise of new technology and the COVID‐19 pandemic, social media (SM) support for breastfeeding has become increasingly popular and it is important to understand how and why mothers access such support, and from whom, to optimise services and to meet mothers’ needs. Increasing research is building on women's use and experience of SM for breastfeeding, although there is a paucity of UK data. This systematic review aimed to understand the impacts of SM support for breastfeeding, including benefits and challenges, to establish the evidence for wider provision within maternity services. The search was limited to studies published in English and focused on the self‐directed use of social media groups for breastfeeding (defined as platforms that facilitate group support via interactivity, allowing for user‐generated content and subsequent responses). Of 327 papers retrieved, 13 studies were included for review. The six themes identified were: breastfeeding context, including factors impacting women's decision making; the relational impact of belonging to an online community; increased self‐efficacy; critiques of SM; the nature and types of support commonly sought and received; and breastfeeding duration as an outcome. The findings confirm that mothers value SM groups for community support, which normalises breastfeeding and provides the support they attribute to improved outcomes, and highlight that UK research focused on provision linked to wider services is needed.

## Key messages

- Joining a social media (SM) group to seek support is common among those mothers intending to breastfeed. For these mothers SM groups have the potential to provide valued support where it is lacking, or in addition to their existing networks.
- SM groups offer reciprocity of knowledge sharing and esteem support. Belonging to a supportive online community promotes emotional wellbeing and self‐efficacy, improving breastfeeding outcomes.
- Online peer support is valued and trusted as a complementary source of information and shared experience. Mothers are aware of issues of reliability, lack of regulation and the need to be discerning of online advice.

## Graphical abstract

With the rise of new technology and the COVID‐19 pandemic, social media (SM) support for breastfeeding has become increasingly popular and it is important to understand how and why mothers access such support, and from whom, to optimise services and to meet mothers’ needs. Thirteen studies were systematically reviewed. The findings confirm that mothers value SM groups for community support, which normalises breastfeeding and provides the support they attribute to improved outcomes, and highlight that UK research focused on provision linked to wider services is needed.

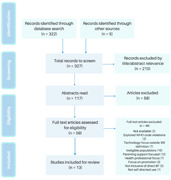

## 1 INTRODUCTION

Breastfeeding is well established as protective of infant and maternal health, but the United Kingdom has some of the lowest breastfeeding rates in the world (McAndrew et al, 2012; Victora et al., 2016). This has serious public health implications, impacting individuals across the lifespan (Indrio et al, 2017). Despite high levels of intention and motivation to breastfeed, women are struggling to meet their breastfeeding goals, with many stopping before they feel ready (Brown, 2019). This current societal failure to support those mothers who want to breastfeed to meet their goals carries economic, psychosocial, and health burdens across generations (Brown, 2019; Rollins et al., 2016; Victora et al., 2016).

Improved education around how breastfeeding works, how to overcome challenges and understanding of normal baby behaviour, alongside practical and emotional support with breastfeeding, is needed—and desired—by new mothers (Brown, 2017). A wide body of research shows that breastfeeding support, delivered by a range of individuals including professional, trained peers and lay supporters, is a key component in meeting the public health responsibility of increasing UK breastfeeding rates. This support works best when it is high quality, consistent and tailored to the setting (McFadden et al., 2017).

Integral to this is peer support; support delivered by a social network of other mothers who have breastfed, sometimes with or without formal breastfeeding support training (Dykes, 2005). Although research examining outcomes of peer support on breastfeeding rates is mixed, often due to inconsistencies in delivery and measurement (Trickey et al., 2018), the research is clear that mothers value peer support (Thomson & Trickey, 2013). It is most effective when delivered in conjunction with professional support across a combination of settings (Ingram, 2013; Sinha et al., 2015). However, due to a combination of funding cuts and COVID‐19, many mothers are increasingly struggling to access face‐to‐face peer support and are frequently turning to online support to fill the gap (Black et al., 2020; Brown & Shenker, 2020; Regan & Brown, 2019).

With the rise of smartphone use and widespread access to social media (SM) platforms (Aichner et al., 2021), SM communities are now central to accessing parenting support. Seventy‐five percent of the global population aged over 13 years are SM users, and Facebook currently has 2.9 billion active monthly users (Data Reportal, 2022), creating a large platform from which to access support and social connection. As a result, most new mothers now use SM to seek advice and believe SM is a beneficial form of support during the transition to parenthood (Baker & Yang, 2018). The need for this support and connection has been heightened during the COVID pandemic, isolating new parents from their existing physical social networks, and preventing the development of new ones (Brown & Shenker, 2021). Although evidence reviews have concluded face‐to‐face support for breastfeeding is most effective, (McFadden et al., 2017), SM functionality and use have changed considerably since the data they examined was collected. As the provision of online breastfeeding support has become more widespread and accessibility has improved, mothers are engaging with it and reporting benefits (Morse & Brown, 2021).

It is important to understand how and why mothers use SM to access breastfeeding support and which mothers find it useful to build on this provision, targeting services effectively. Scoping searches identified several systematic reviews that have provided insight into the evidence available. However, these have focused on internet‐based ‘interventions’ in general (Almohanna et al., 2020; Giglia & Binns, 2014), breastfeeding outcomes (Orchard & Nicholls, 2020) or on specific populations, for example, pregnant women only (McArthur et al., 2018). The findings highlight that interactivity and personalisation are key to successful internet‐based interventions (Almohanna et al., 2020) and that they are a viable option for breastfeeding advocacy (McArthur et al., 2018), particularly if used in combination with and to augment standard care (McArthur et al., 2018; Orchard & Nicholls, 2020). Notably, none focused on the evidence in relation to women's experiences of SM groups as a medium for community breastfeeding support.

This systematic review aims to identify the existing evidence in relation to SM group use for breastfeeding support, why mothers access such support and from whom, to optimise services and to meet mothers’ needs. The purpose of this systematic review is therefore to:

1. To establish the existing evidence on the use of SM groups/communities for breastfeeding support.
2. To identify any reported benefits, challenges and impacts of accessing SM group/community support for breastfeeding.

To keep the review focused, the following research question was used: What are the impacts of SM group use for breastfeeding support?

## 2 METHODS

To address the research questions through the identification of key terms and synonyms a search strategy (Table 1) and eligibility criteria (Table 2) were designed, modifying the Population, Issue, Context, Outcome (PICO) tool (Fineout‐Overholt & Johnson, 2005). This was modified to include both Issue (qualitative) and Intervention (quantitative) terms, to capture the most comprehensive range of results (Aveyard et al., 2016).

**Table 1:** PICO tool (Boolean operator OR)

	Population	Issue/intervention	Context	Outcome
PICO term	Breastfeed*	Social Media	Support	Experience
Alternatives/synonyms	Infant feeding	Facebook	Continu*	Duration
				Breastfeed*
	Post‐natal	Online		Perception*
	Mother	Social network*		
	Pregnan*	Communit*		

*Note*: * indicates a truncation enabling database searching of the main stem of the word.

Abbreviation: PICO, Population, Issue, Context, Outcome.

**Table 2:** Eligibility criteria

Inclusion criteria	Original research article
	Written in English
	Studies focused on social media (as per chosen definition*)
	Studies focused on self‐directed social media use for support with direct breastfeeding
Exclusion criteria	Written in another language
	Studies focused on other populations, for example, not those currently breastfeeding
	Studies focused on social media use for wider parenting support
	Studies limited to support for exclusive expression only
	Studies focused on social media use for breastfeeding promotion rather than support
	Studies limited to health professional input to the exclusion of peer support
	Studies focused on technology outside the identified definition of social media
	Studies focused on social media as a controlled intervention

*Note*: * indicates a truncation enabling database searching of the main stem of the word.

### 2.1 Eligibility criteria

Published and unpublished studies meeting the inclusion criteria (Table 2) were eligible. No geographical limits were set to ensure as broad a review as possible. While acknowledging any demographic differences that may impact generalisability, it was considered inclusion would reduce bias and unfairly skewed data (Van Aert et al., 2019).

Although there is no definitive definition of SM, for the purposes of this review SM is limited to platforms which facilitate group support via interactivity, allowing for user‐generated content and subsequent responses. This includes online web‐based message board communities (e.g., Babycenter, Mumsnet), but excludes specific app‐only technologies, due to their limited, targeted use. No date limits were set to capture all relevant studies, recognising that the definition of SM would apply restrictions to dates in relation to its inception. The broadly agreed date for the inception of these platforms, using the definition of SM as virtual communities, is 1997 (Aichner et al., 2021). Facebook (founded in 2004, 1.93 billion daily active users), Twitter (2006, 174 million) and Instagram (2010, 500 million) are the three leading platforms (Alhabash & Ma, 2017; Statista, 2021).

Eligibility criteria were developed with a second reviewer to reduce bias and included studies checked by both reviewers against the criteria set. However, improving interrater reliability through both reviewers conducting the literature search was not possible as this systematic review forms part of a thesis, requiring flexibility (Siddaway et al., 2019).

### 2.2 Search strategy and screening

Literature was sought from October to November 2021. Scoping searches highlighted a focus on intervention outcomes rather than experiences in previous reviews and a need to set clearly defined search limits to identify relevant studies. As a result, 16 search terms were used in various combinations using Boolean operators (Table 1), for example, (breastfeed* OR infant feeding) AND (social media OR Facebook) AND (Midwi* OR health professional) AND (support OR promot*). ASSIA, CINAHL, PubMed/Medline, ProQuest, MIDIRS, EBSCOHost, Scopus, Google Scholar and iFind were searched using these terms. A total of 322 published and unpublished studies were identified, and an additional five through reference list searching of relevant books and articles to minimise any exclusions.

**Figure 1:**
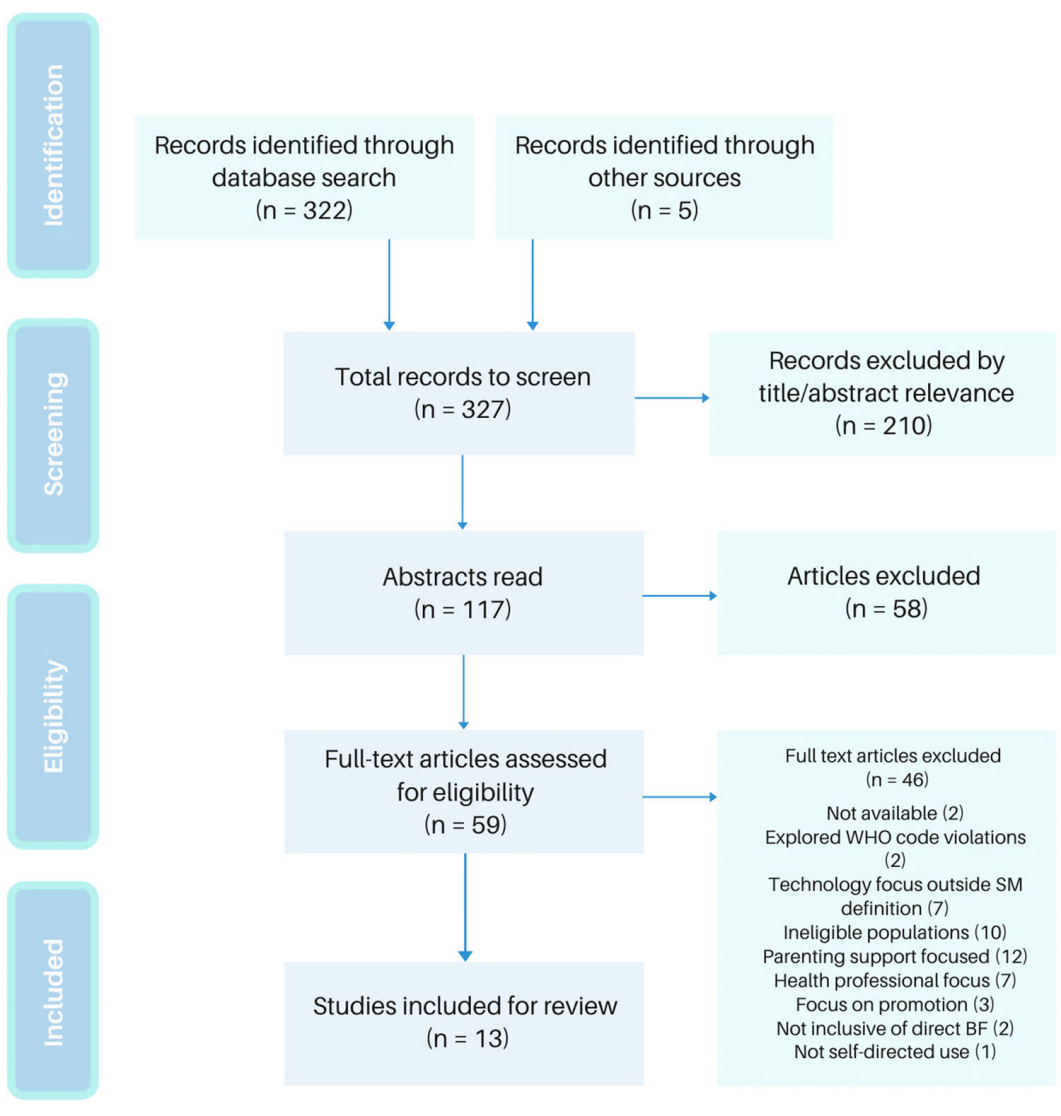
PRISMA flow diagram demonstrating the article screening process. PRISMA, Preferred Reporting Items for Systematic Reviews and Meta‐Analyses; WHO, World Health Organisation.

All records were screened, identifying that despite the presence of relevant search terms, many studies were focused on breastfeeding promotion or the use of specific digital interventions (such as mobile apps). After initial exclusion for relevance, 117 abstracts were read and the eligibility criteria applied, leaving 59 full‐text articles. Forty‐six articles were excluded. The excluded studies included those which focused on offering SM support as a specific intervention (where results may not be comparable to those who interact with self‐directed groups) and those which related only to specific populations (e.g., preterm infants), which focused on exclusive pumping support only. The majority of those excluded were from studies where the SM support group was related to parenting in general, rather than primarily being focused on breastfeeding support (see Figure 1 for the full list of reasons for exclusion). Thirteen articles remained for review.

### 2.3 Data extraction

A data extraction form was adapted (Aveyard et al., 2016) to summarise the study characteristics, findings, strengths and limitations and to aid the analysis of the 13 remaining studies (Appendix A). These were also appraised using published critical appraisal checklists. Nine qualitative studies (Appendix B) were analysed using the applicable Critical Appraisal Skills Programme UK (CASP UK) checklist (Critical Appraisal Skills Programme UK, 2018). The final five studies used mixed methods approaches and were analysed using the Quality assessment with diverse studies (QuADS) criteria (Harrison et al., 2021), particularly enhanced and refined for use with health service research (Appendix C).

In total, the 13 studies (8 qualitative and 5 mixed methods studies) represented a total sample size of 507 mothers, and the analysis of 2767 SM posts. They were conducted between 2015 and 2020, reflecting widespread smartphone use. A recent upsurge in research activity in this area was notable, with 10 studies clustered between 2019 and 2020. Studies were conducted in New Zealand (1), the United States (6), Australia (2), the United Kingdom (2) and Ireland (2). Although comparisons can be drawn demographically between these research locations, care was taken to acknowledge the differing cultural and social contexts (including attitudes and breastfeeding rates) in relation to breastfeeding support. Of the UK studies, one involved a small sample (*n* = 12) and the other (*n* = 12), although a large number of group posts (*n* = 1230) were also included in the analysis. The latter was also 6 years old, so a lack of current UK literature was notable, particularly in the pandemic context and surge of SM use.

### 2.4 Data synthesis

To address the research question underpinning this review (What is the impact of SM group use for breastfeeding support?), a modified narrative synthesis approach was taken (Popay et al., 2006), using three stages. This approach was considered appropriate to identify common themes across the literature, although these do not all relate directly to the data collected in each study (Braun & Clarke, 2014). First, after familiarisation with the studies, initial codes were produced using NVivo v12, identifying themes via inductive thematic analysis. Second, themes were reviewed in relation to the coded extracts, which were then defined and named. Third, the robustness of the synthesis was evaluated independently by a second reviewer (Popay et al., 2006).

A reflexive journal was used to reflect on methodological decisions and the reviewer's background in breastfeeding support and influences as a health professional. Confidence in the findings was developed via both prolonged engagement with and persistent observation of SM groups before the review.

## 3 RESULTS

### 3.1 Study quality

All the studies explored SM use for breastfeeding support, with seven including analysis of impacts on breastfeeding outcomes/duration (Black et al., 2020; Herron et al., 2015; Robinson, Davis, et al., 2019; Robinson, Lauckner, et al., 2019; Skelton et al., 2018; Wilson, 2020) (see Table 3). The studies all had clearly defined aims and recruitment strategies and noted the breastfeeding context (including demographic and sociocultural background) as confounding factors in drawing conclusions on the impact of SM group support. Overall, there were a few issues with study quality. Although sample sizes were generally small, this was expected for the qualitative methods, generating rich insights from individuals (Braun & Clarke, 2014). These were confirmed by content analyses of large numbers of SM posts, enhancing the findings (Snelson, 2016). Most of the studies commented on the potential reflexivity issues arising from research done by those with direct connection to the SM group being studied (in some cases as a midwife or breastfeeding counsellor).

**Table 3:** Summary of strengths and limitations of studies

Authors/year	Strengths	Limitations	Population	Design
Alianmoghaddam et al. (2018)	Discusses wider social contexts influencing breastfeeding practices and focuses on quality Methods employed multiple strategies (survey, face‐to‐face and monthly telephone interviews) enriching data Several theoretical constructs were discussed and applied	Small, homogeneous sample (*n* = 30) and research location limits generalisability Samples were highly motivated with the intention to breastfeed for at least 6 months	30 mothers breastfeeding babies 0–6 months (New Zealand)	Qualitative
Black et al. (2020)	Approach explores socioeconomic, cultural and individual factors alongside mothers’ perceptions Detailed exploration of the theoretical lens (social cognitive theory) and possible value in the analysis	Research limited to members of one group in one research location Homogeneous, small sample, all partnered/married with one child, limiting generalisability	8 women from one FB group (Ireland)	Qualitative
Bridges et al. (2018)	Offers insights into type and usefulness of support, including from whom Adds detail on commonly discussed topics Methods captured a large sample of posts and comments and included shared images	Researcher status as ‘insider’ may impact reflexivity Focuses on perceptions of a supportive community, no data on impacts on breastfeeding No demographic data were captured, all groups were run and moderated by the same organisation, which may limit generalisability	778 wall posts with a total of 2998 comments (Australia)	Online ethnography (Qualitative)
Skelton et al. (2018)	Demonstrates clear positive influence of social media support on attitudes, knowledge and behaviour Combination of methods resulting in aggregated data for analysis Adds insight into groups as a resource and a community and impact on outcomes	Research limited to members of one group in one research location Homogeneous, small sample, limiting generalisability Included reflections from mothers who had stopped breastfeeding up to 3 years prior, so some data was retrospective/subject to recall bias	21 women (focus group) and 12 mothers (interviews) from one FB group (US)	Qualitative
Skelton et al. (2020)	Detailed discussion of underpinning theoretical constructs, and identifying clear characteristics of a CoP Relatively large sample drawn over both approaches	Homogeneous, highly motivated sample Cross‐sectional design limits the determination of causality	21 women (focus group) and 12 mothers (interviews) from one FB group (US)	Cross ‐sectional
Robinson, Davis et al. (2019)	Adds insight into the needs of a specific population Detailed discussion of underpinning theoretical constructs Detail is provided on the correlation between independent variables and breastfeeding duration	Potential selection bias, design limits determination of causation Limited generalisability due to demographics and large FB group size	277 African‐American mothers from 9 FB groups (US)	Cross‐sectional
Bridges (2016)	Both administrators and mothers participated Provides detail on the range of ‘added value’ of online support alongside traditional formats Details perceptions of information reliability	Researcher status as ‘insider’ may impact reflexivity No demographic data on participants collected Small sample (*n* = 23), specific group formats and moderation by ABA‐trained supporters may impact generalisability	3 FB groups were observed, followed by 23 group participants interviewed (Australia)	Qualitative
Regan and Brown (2019)	Well‐designed study meeting all Critical Appraisal Skills Programme UK (CASP) (2018) checklist criteria Highlights drawbacks in addition to benefits Explores support sources/group moderation	Limited (*n* = 14), homogeneous and highly motivated sample Most had previous experience of breastfeeding	14 mothers breastfeeding child up to 3 years (UK)	Qualitative
Lebron et al. (2020)	Systematic, rigorous analysis using iterative methods Analyses both questions and responses, offering insight into information sharing without constraint	Demographic data largely unknown International forum/message board limits generalisability to other SM platforms No data on behavioural impacts/breastfeeding impacts Limited to one forum and peer‐only support	258 posts and 1445 corresponding comments (US)	Content analysis
Wagg et al. (2019)	Consideration is given to online community context and significance Useful insight into support‐seeking behaviours Confounding variables discussed	Data collected over a small timeframe (7 days) No examination of post quality, experiences or perceptions	501 posts and associated comments. Most from mothers with babies 6 weeks–6 months (UK)	Content analysis
Robinson, Lauckner et al. (2019)	Well‐designed study meeting all CASP (2018) checklist criteria Adds detailed perspectives for this population of mothers not included elsewhere Detailed discussion of theoretical lens Includes data related to critique of groups in addition to positive perceptions	Potential selection bias Generalisability may be limited to the sample demographics Cross‐sectional design may impact generalisability	22 Black mothers (US)	Qualitative
Herron et al. (2015)	Phased mixed methods approach adds to rigour and validity of the analysis Includes impacts on outcomes and detailed discussion of factors relating to reciprocity	Demographic data are not available Forum/message board limits generalisability to other SM platforms Data collected <10 years ago	1230 online messages, online interviews with 12 women (Ireland)	Mixed methods concept analysis
Wilson (2020)	Methods enable exploration of social support and modifiable factors over time Includes detailed discussion of theoretical constructs Development of predictive model offers framework for future research	Samples were <1 month post‐natal at the time of the first survey and >6 months for the second survey, so responses were subject to endurance, concentration and time factors for large surveys (high attrition rate) No detail on perceived credibility or quality of groups	241 women from 17 FB BF groups 1230)(US)	Longitudinal mixed methods

Abbreviations: ABA, Australian Breastfeeding Association; BF, breastfeeding; CoP, community of practice; FB, Facebook; SM, social media.

The two papers by Skelton et al. (2020, 2018) analyse the same data using different methods. Thematic analysis is used to identify themes in relation to mothers’ use and experience of accessing support via a single Breastfeeding Support Facebook (BSF) group (Skelton et al., 2018). The subsequent paper (Skelton et al., 2020) uses inductive content analysis to analyse the same interview and focus group data, iteratively guiding a second quantitative phase of the study through a theoretical lens. This has strengths and limitations. The mixed methods and sequential analyses provide detailed insight into the BSF group's function as a community and mothers’ perceptions, enabling their conceptualisation as online communities of practice. Despite results relating to the same data, both papers were included to reflect the additional insights.

Similarly, Robinson, Davis et al. (2019) and Robinson, Lauckner et al. (2019) present two papers that form part of one larger study, although different data sets are analysed and discussed, avoiding ‘double counting’. Robinson, Davis et al. (2019) and Robinson, Lauckner et al. (2019) collected and analysed quantitative survey data to explore the relationship between BSF group support, outcomes and self‐efficacy and strengthen these findings using thematic analysis of focus group data. Two further papers (Bridges, 2016; Bridges et al., 2018), while relating to two separate studies, also involve the same lead author. While this approach provides rich data and triangulation of findings, it should be noted that multiple papers from the same authors may impact the breadth of the review.

### 3.2 Study themes

Six themes were identified from the 13 included studies: breastfeeding context, including sociocultural antecedents and individual factors impacting women's decision making; the impact of belonging to an online community, relating to the virtual relationships underpinning the impact online support; increased self‐efficacy; critiques of SM support; the nature and types of support commonly sought and received; and breastfeeding duration as an outcome. Seven studies mentioned all six themes and a further two contained five of the six (Table 4). All the studies recognised the significance of the context in which women breastfeed (Theme 1), and the function of the SM support group as an online community. The nature of support available via SM groups, and on which topics (Theme 5), was discussed by all studies. Self‐efficacy was also a prevalent theme, with 13 studies identifying the impact of access to SM support on women's belief in their own capacity to achieve their breastfeeding goals as a predictor of improved experiences and outcomes.

**Table 4:** Contribution of each study to themes

Theme 1: The impact of SM group support on the breastfeeding context	Alianmoghaddam et al. (2018), Black et al. (2020), Bridges (2016), Bridges et al. (2018), Herron et al. (2015), Regan and Brown (2019), Robinson, Davis et al. (2019), Robinson, Lauckner et al. (2019), Skelton et al. (2018, 2020), Wagg et al. (2019), Wilson (2020)
Theme 2: The impact of belonging to an online community	Alianmoghaddam et al. (2018), Black et al. (2020), Bridges (2016), Bridges et al. (2018), Herron et al. (2015), Lebron et al. (2020), Regan and Brown (2019), Robinson, Davis et al. (2019), Robinson, Lauckner et al., (2019), Skelton et al. (2018, 2020), Wagg et al. (2019), Wilson (2020)
Theme 3: Increased self‐efficacy	Alianmoghaddam et al. (2018), Black et al. (2020), Bridges (2016), Bridges et al. (2018), Herron et al. (2015), Robinson, Davis et al. (2019), Robinson, Lauckner et al. (2019), Skelton et al. (2018, 2020), Wagg et al. (2019), Wilson (2020)
Theme 4: Issues arising from SM support for breastfeeding	Alianmoghaddam et al. (2018), Black et al. (2020), Bridges (2016), Bridges et al. (2018), Herron et al. (2015), Regan and Brown (2019), Robinson, Davis et al. (2019), Robinson, Lauckner et al. (2019), Skelton et al. (2018, 2020)
Theme 5: Nature of support and topics	Alianmoghaddam et al. (2018), Black et al. (2020), Bridges (2016), Bridges et al. (2018), Herron et al. (2015), Lebron et al. (2020), Regan and Brown (2019), Robinson, Davis et al. (2019), Robinson, Lauckner et al. (2019), Skelton et al. (2018, 2020), Wagg et al. (2019), Wilson (2020)
Theme 6: Breastfeeding duration	Alianmoghaddam et al. (2018), Black et al. (2020), Herron et al. (2015), Robinson, Davis et al. (2019), Robinson, Lauckner et al. (2019), Skelton et al. (2018, 2020), Wilson (2020)

Abbreviation: SM, social media.

### 3.3 Theme 1: The impact of SM group support on the breastfeeding context

Women's experiences of breastfeeding within their family and in a wider sociocultural context are a significant factor in the initiation and continuation of breastfeeding (Rollins et al., 2016). All but one study (Lebron et al., 2020) highlighted context as a confounding factor that cannot be controlled for, and findings should be considered in this context, particularly when considering whether there is any association between SM group use and breastfeeding duration. Several theoretical approaches were applied by the studies to understand the significance of women's sociocultural context on breastfeeding behaviours and whether SM group use mediates this effect.

It is well established that social support for breastfeeding (including ‘significant others’ or ‘strong ties’ such as partners, close family members and friends) and living and working within a culture that respects breastfeeding, and a society that facilitates and supports it, are key to women's decision making and success (Brown, 2017; Tarkka et al., 1999). Many women do not have access to adequate or consistent support through their existing networks (Wilson, 2020). Applying Milligan and Wiles (2010) theory of ‘landscapes of care’, which argues that social and emotional support can be geographically distant but remain proximate, Alianmoghaddam et al. (2018) highlight the impact of digital communication on the cultural and social contexts of a mother's life. They found that mothers’ breastfeeding knowledge and behaviour are influenced by family members, positively and negatively, via SM communication despite not being physically present. However, exclusive breastfeeding is also shaped by the social network of ‘weak ties’ accessed by mothers via SM support groups, which promotes, normalises and supports breastfeeding continuation via the circulation of information. As such, the social context in which women breastfed was altered by their membership in the online community (Alianmoghaddam et al., 2018).

Using social cognitive theory as a framework, which asserts that behaviour depends on the interplay between women and their environment (Bandura, 1997), Black et al. (2020) found that women who belonged to an SM support group were influenced by their ability to provide social and emotional support. They reported group use incentivised continued breastfeeding compared with reliance on existing social support. Robinson et al. (2019) applied the integrated model of behaviour prediction (IMBP) to SM support group use, focusing on intention as the strongest predictor of outcomes, itself determined by attitude, norms and agency (Montano & Kaspryzk, 2015). They found that in comparison to other support sources, support from a Facebook group was significantly correlated with intended breastfeeding duration. Women received more support from SM than from family and friends for breastfeeding (Robinson, Lauckner, et al., 2019) strengthening the evidence that the sociocultural context underpinning women's breastfeeding choices and behaviour can be mediated or reinforced by SM support.

Wagg et al. (2019) also frame their findings (that SM groups effectively facilitate support seeking) within social support theory, highlighting the impact of the collective context, where shared experience and a shared language are fundamental to accomplishing goals. This mediates the effect of a lack of breastfeeding experience, support and knowledge within a woman's existing ‘strong tie’ network (Alianmoghaddam et al., 2018; Herron et al., 2015). All the studies found that the breastfeeding context played a critical role in women's intentions, experiences and breastfeeding outcomes and that this could be mediated by the SM group support.

### 3.4 Theme 2: The impact of belonging to an online community

Eleven studies focused on Facebook groups, two explored message board platforms (Babycenter and Netmums), and one included all types of SM. Although both Facebook and online web‐based message boards facilitate group support, differences in how they function as communities should be noted. An online community is defined as a social network of interactions between members who come together online with a shared purpose (De Souza & Preece, 2004) and group size; interactivity and responsiveness are antecedents of sustainable online communities (Dover & Kelman, 2018). As interactions within these communities differ across the platforms, with Facebook groups being more widely used and more frequently engaged with (Alianmoghaddam et al., 2018), care should be taken when synthesising findings.

However, all the studies identified the formation and function of an online community, and its accessibility, as significant to the impact of SM support on women's breastfeeding experiences and outcomes. Their findings noted the positive impact of belonging to a supportive online community on psychosocial factors such as emotional wellbeing and self‐efficacy, as well as on breastfeeding outcomes (Black et al., 2020; Robinson, Davis, et al., 2019). Women choose to become members of groups that meet their interpersonal and informational needs, seeking practical, social and emotional support from those with shared or lived experience (Bridges, 2016; Bridges et al., 2018; Regan & Brown, 2019). Notably, women choose which groups will meet their needs based on a belief that they belong within the social group it represents. Robinson, Davis et al. (2019) and Robisnon, Lauckner et al. (2019) found this was particularly important for African‐American women who did not feel represented elsewhere. Other studies found that their participants were homogeneous and highlighted the significance of shared culture and goals in creating a cohesive and growing community, alongside the limitations of the medium in reaching other population groups. Evidence for the UK peer support provision is limited by a lack of diversity among samples, where White, older and educated mothers are more likely to have taken part in studies (McFadden et al., 2017), providing little insight into the needs of other groups or how to improve provision.

Mothers felt having easy access to a supportive community and a sense of belonging had an overall positive impact on them and their breastfeeding goals, emphasising feelings of empowerment, shared experience and solidarity (Wagg et al., 2019; Wilson, 2020). In addition, the community functions as a developing resource, hosting factual and experiential information, which provides reassurance, increases confidence and influences parenting decisions and behaviours (Bridges et al., 2018).

Skelton et al. (2020) conceptualise SM groups as online communities of practice, identifying key characteristics including skill‐building and the development of trust through interaction.

The development of group norms was a prevalent finding within the community theme, the key to developing a sense of belonging that resulted in extended breastfeeding goals (Black et al., 2020; Wagg et al., 2019) validation and a sense of identity (Wagg et al., 2019). The development of a community, creating networks of mothers with shared experiences and purpose has a positive effect on both wellbeing and breastfeeding outcomes. Little data was collected in relation to differences between mother‐to‐mother, trained peer support and professional support, although Herron et al. (2015) highlighted variations in Netmums thread dynamics, with reduced engagement with posts from professionals. On Facebook groups associated with breastfeeding organisations, trained peer support motivated engagement (Bridges, 2016) and trust, playing a vital role in moderating discussion that positively impacted the community (Bridges et al., 2018).

### 3.5 Theme 3: Increased self‐efficacy

Self‐efficacy or women's belief in their capacity to achieve their goals is well established as a predictor of breastfeeding success and satisfaction (Awaliyah et al., 2019). Ten studies noted the impact of SM group use on the sense of agency and empowerment reported by participants. Within the online community, breastfeeding is perceived as normal and desirable, and solutions are offered to challenges that reinforce ongoing goals as achievable (Black et al., 2020). For mothers, reading about the successful experiences of others offers encouragement and the sharing of skills offers support to overcome challenges (Robinson et al., 2019). This is particularly evident within a sociocultural context of low breastfeeding rates: few women are supported with lived and shared experiences by close friends and family and many experience the recommendation to switch to formula feeding as a solution to practical and emotional challenges. Where family support is available, it may contradict current evidence‐based recommendations, and women seek clarification from peers online (Alianmoghaddam et al., 2018). This is particularly important for increasing breastfeeding self‐efficacy among groups with lower breastfeeding rates and greater perceptions of breastfeeding barriers (Robinson et al., 2019).

Mothers who choose to become and remain members of breastfeeding SM groups perceive them as empowering, encouraging active participation in decision making through the provision of health information (Bridges, 2016). Easy access to this information and peer support increases confidence in a woman's ability to manage problems and make decisions, which in turn increases breastfeeding rates (Bridges, 2016; Sheehan et al., 2009). Robinson et al. (2019), through the IMBP theoretical construct of ‘personal agency’, describe how self‐efficacy and a woman's perception of how much control she has over her ability to breastfeed are positively impacted by the influences of the online community.

The community also plays an important role in increasing self‐efficacy and self‐esteem by offering the opportunity to help others, and women are keen to share in a sense of community and connectedness through reciprocity (Bridges, 2016; Skelton et al., 2020). This is a critical therapeutic process within support groups (Pagano et al., 2010), increasing self‐efficacy through altruistic ‘paying forward’ of support, and was a key theme across all studies. Herron et al. (2015) identified indirect reciprocity as a pivotal component of the model of online breastfeeding support they propose, highlighting the ways in which women helped and supported one another, returning to the group to share information and support with others. The ability to overcome challenges, and to share solutions with others, generates greater self‐efficacy, extending breastfeeding duration (Black et al., 2020) and this is a key function of the SM group as a community of practice.

Wagg et al. (2019) also highlight the role of ‘esteem support’ within the group in promoting self‐efficacy, noting the prevalence of responses offering encouragement, expressions of pride and words of congratulations. Significantly, esteem support was second only to informational support in the type of support requested (Wagg et al., 2019) providing a ‘circle of peer support’ with an overall positive effect on confidence and self‐esteem (Regan & Brown, 2019).

### 3.6 Theme 4: Issues arising from SM support for breastfeeding

Ten studies highlighted concerns in relation to SM breastfeeding support. The most common of these was the reliability of information available within groups, an issue regularly highlighted in the wider literature (Ellis & Roberts, 2019), although the generation of women widely using SM for health and parenting support generally view it as a reliable source (Morse & Brown, 2021). The findings of the studies reviewed suggest that women who belong to online breastfeeding support communities felt real‐time information from peers with lived experience was a valid and reliable resource, often trusting this over advice from healthcare professionals (Skelton et al., 2018), and use it to compensate for poor support elsewhere (Robinson et al., 2019). However, women are aware that information on SM is unregulated, sometimes impacting their confidence in the advice (Regan & Brown, 2019). Women acknowledged the need to be discerning, particularly in relation to medical advice (Regan & Brown, 2019), and that this ability develops as they become ‘expert’ themselves (Herron et al., 2015), but is also dependent on women's general digital and health literacy (Alianmoghaddam et al., 2018). Online self‐correction may also occur, where inaccurate postings are promptly corrected through teamwork from the within message threads (Herron et al., 2015) and online communities (Skelton et al., 2020), potentially increasing their reliability as a resource.

Trust in the reliability of information and in the motivations of others exists where connection and rapport develop as a result of empathetic facilitation and support styles (Bridges et al., 2018). Mothers report seeking a wide variety of opinions on an issue to direct their decision making (Robinson et al., 2019) and this growing trust in the community, and reliability of the advice is key to the adoption of recommendations and goal setting (Black et al., 2020; Skelton et al., 2018). However, these findings may be impacted by the demographics of those who are self‐motivated to seek online support, with overrepresentation of more affluent and highly educated women within the samples. All studies recognised this as a limitation.

Polarised debate and experiences or fear of judgement were also reported (Herron et al., 2015; Regan & Brown, 2019). On message boards, differentiation was found between ‘support’ and ‘debate’ threads, with the latter often expressing negative sentiment reflecting public discourse, rather than positive support. This was largely regarded as an opportunity for discussion, resulting in becoming politically aware and developing confidence in parenting decisions and philosophies (Bridges, 2016; Herron et al., 2015). However, judgement, conflicting advice and polarisation had negative impacts for some, a key finding in developing insight into the wider experiences of women using SM support (Herron et al., 2015; Regan & Brown, 2019).

### 3.7 Theme 5: Nature of support and topics

Four studies used methods that involved the direct analysis of online posts (Bridges et al., 2018; Herron et al., 2015; Lebron et al., 2020; Wagg et al., 2019). Identifying similarities in the content and motivations for online posts, their findings were consistent: women turn to SM group support both where their access to face‐to‐face support is inadequate and to complement this support. They seek information most often statistically, but emotional and esteem support (encouragement and reassurance) are significant (Wagg et al., 2019). These findings are supported by the other studies; women value, seek and benefit from online interaction, giving and receiving social and emotional support alongside knowledge sharing (Black et al., 2020; Skelton et al., 2020). Emotional and esteem support result in increased confidence, self‐efficacy and empowerment, with positive impacts on breastfeeding outcomes and experience (Bridges, 2016).

Looking at common topics, the studies found that queries generally related to breastfeeding management (including physical and practical management such as positioning, attachment and feeding frequency), health (including mother and baby, physical and mental health) and the breastfeeding journey (including work‐related queries, feeding in public and parenting philosophies) (Bridges et al., 2018; Wagg et al., 2019; Wilson, 2020). The specific topics women seek support for correlate with the most common breastfeeding problems, which lead to early cessation (Bridges et al., 2018). Informational support relating to the physiology and management of breastfeeding is evident as a clear need, not being effectively fulfilled elsewhere, including by professionals (Regan & Brown, 2019; Skelton et al., 2018). It is clear, however, that women are also seeking to fulfil emotional support needs, including reassurance about what is normal and solidarity in the breastfeeding journey, and to reduce social isolation (Regan & Brown, 2019; Skelton et al., 2020). This is achieved via information seeking, sharing and giving, centred on previous knowledge and experience, alongside encouragement to continue (Lebron et al., 2020). Sustained breastfeeding duration was linked to a positive attitude derived from greater knowledge and confidence (Wilson, 2020).

Another key finding was that many of these social and informational benefits can also be derived from ‘lurking’ (reading posts without interacting). High levels of passive viewing were observed, offering mothers the opportunity to observe and learn at a level of anonymity that suited their needs and circumstances (Herron et al., 2015). This behaviour was also influenced by group dynamics and culture, including how a woman felt her query would be received (Robinson et al., 2019). This changed over time: with time and breastfeeding experience, women posed fewer questions but answered more (Robinson et al., 2019), developing a community of practice through joint problem solving and reciprocity (Skelton et al., 2020).

Several studies also commented on the potential impact of group moderators on group function and support, noting their significance for correcting misinformation (Regan & Brown, 2019; Skelton et al., 2020) and modelling an empathetic approach to providing support (Bridges et al., 2018).

### 3.8 Theme 6: Breastfeeding duration

Breastfeeding duration as an outcome was explored by eight studies. All noted that direct causation cannot be determined due to the complexity of the breastfeeding context and the impossibility of controlling for confounding factors. However, they conclude that SM group use is a variable in sustained breastfeeding, through influence on breastfeeding knowledge, attitudes and behaviours (Skelton et al., 2020), increased self‐efficacy (Black et al., 2020) and receipt of emotional support (Bridges, 2016), which may result in extended goals (Black et al., 2020) and duration (Robinson et al., 2019). There were also impacts noted on wider parenting practices (Herron et al., 2015) and philosophies associated with extended breastfeeding duration, such as babywearing or bedsharing (Bridges, 2016).

One study found half of the women who seek online support in SM groups to initiate or continue breastfeeding continue to do so weeks and months later (Herron et al., 2015). This may reflect the motivation of those who seek help but also suggests a positive impact of a group membership. The normalisation of breastfeeding and related behaviours within the online community was also noted, with an impact on the breastfeeding goals women set, extending what they felt was achievable and desirable (Black et al., 2020). Motivation is a key antecedent of breastfeeding success, and by seeing others succeed, group members are motivated through increased self‐efficacy to extend their goals (Black et al., 2020; Robinson et al., 2019) beyond wider social norms. Robinson et al. (2019) found an average intended breastfeeding duration of 18.9 months and a significant relationship between intended duration and Facebook support. The sample studied by Skelton et al. (2020) also reported higher initiation rates, exclusivity and longer duration than the national average.

Confidence, knowledge and attitude are significant predicators of breastfeeding duration. Wilson (2020) found that these variables can be modified by SM group use, resulting in sustained exclusive breastfeeding at 6 months. The strength of social support available was also significant, with women continuing to breastfeed beyond 6 months more likely to describe their social support, including from the SM group, as positive (Wilson, 2020).

Evidence suggests that provision with a wide delivery context, enabling mothers to individualise the support they receive based on cultural, social and clinical need, and convenience, is best received and most valued (Trickey et al., 2018). SM groups do this by extending the reach of breastfeeding support provision beyond standard care from maternity and health services, providing access when needed throughout the breastfeeding journey. The mothers’ studied attributed this support to longer breastfeeding duration and improved experiences (Skelton et al., 2018).

## 4 DISCUSSION

This review aimed to establish the existing body of literature relating to the impact of women's self‐directed use of SM groups for breastfeeding support, to identify gaps in knowledge and inform future research. There were some challenges in conducting the search: definitions of SM vary, with the care needed not to exclude relevant studies while ensuring commonalities in the area being explored. Larger randomised controlled and quantitative studies were identified related to interventions or specific populations so were ineligible for inclusion. The studies included rely largely on qualitative findings and smaller samples, which although providing rich data and common themes, limits generalisability and recommendation for investment: outcomes cannot be definitively proven.

However, six themes were identified from the literature, relating to the impacts on breastfeeding context, self‐efficacy and breastfeeding duration, of community membership, the nature of support and common issues. The themes highlight SM support group membership as a strategy for increasing positive breastfeeding experiences, enhancing knowledge, social connections and potentially increasing breastfeeding duration. Most mothers studied perceived belonging to or using an SM group for breastfeeding support as improving their confidence, self‐efficacy and empowerment, resulting in extended breastfeeding goals. The online community was viewed as a safe, supportive space where solutions to breastfeeding challenges are available as and when needed, alongside encouragement, and achieving goals can be celebrated. Many women do not have access to or experience this in a ‘real‐life’ setting.

Strategies to improve breastfeeding continuation rates are needed to support individual women to meet their goals and to enhance public health (Brown, 2017). Self‐directed SM group use is viewed as convenient and accessible by the current generation of women, and their use for health‐related and parenting support needs is widely seen as both normal and acceptable (Alianmoghaddam et al., 2018). Women are turning to online communities to fill the gap created by geographic family dispersal (Alianmoghaddam et al., 2018), a lack of breastfeeding knowledge in existing social networks (Bridges, 2016) and the underresourcing of face‐to‐face services (Regan & Brown, 2019). Those who seek and engage with this form of support find value in a community which normalises and celebrates breastfeeding, providing informational, social and emotional support, which they perceive to result in extended goals and duration. As such, SM appears to provide an ideal, near‐universal and cost‐effective platform for widening breastfeeding support and improving outcomes (Wilson, 2020).

However, while 10 studies identified potential issues relating to the reliability and regulation of online breastfeeding support, just three commented on the potential impacts of trained peer support and/or health professional input (Bridges, 2016; Bridges et al., 2018; Herron et al., 2015). They conclude that there are differences in how information is received, with Bridges (2016) and Bridges et al. (2018) identifying the positive impact on trust and perceptions of reliability when SM groups are facilitated by a breastfeeding organisation comprising of trained supporters/professionals. Herron et al. (2015) found lower engagement on message boards with professional posts. Regan and Brown (2019) note variability in group moderation and the concerns and challenges this presents to mothers, but there is a paucity of research in this area, with no specific evidence of the function, types or impact of SM group moderation on online breastfeeding support communities.

Although some comparisons can be made, the studies also span disparate healthcare systems, in the US, Australia, New Zealand and the UK. As reported by the studies, the context in which women breastfeed, medical and sociocultural, including varying breastfeeding rates, is a significant factor in breastfeeding attitudes and behaviour. Therefore, there may be a global variation that could impact findings and generalisability, including limited or lack of access to SM and technology.

### 4.1 Limitations of this review

Although inevitable as part of a PhD study, a major limitation of this review is that it was conducted by a single reviewer. However, the process was made more rigorous by a second reviewer checking the criteria used and is small enabling both to become familiar with the studies analysed. Both reviewers reviewed the themes and an agreement was reached.

To enable findings to be analysed across comparable self‐directed group use, eligibility criteria were narrowed to exclude groups aimed only at specific populations, for example, those with preterm babies, or exclusively expressing. Although this ensured the findings can be compared and synthesised with greater confidence, it may exclude some further insights. Future reviews could consider specialised groups and groups developed as interventions.

Excluding interventions also limits the sample to those women who are motivated to engage in self‐directed SM group use and those who find it beneficial and remain group members. Content analysis aside, it also limits insights to those willing to take part in studies. It is therefore unknown how impacts of SM support may differ among less motivated or less digitally literate/engaged samples.

The studies tended to have homogeneous samples—predominantly White, married or partnered women with a high level of education. This is representative of the higher prevalence of breastfeeding and digital literacy among this population (Bartick et al., 2017). Robinson, Davis et al., (2019) and Robinson, Lauckner et al. (2019) examine Facebook group support use specifically among African‐American mothers, with a mixed‐income range and of whom a greater proportion was single or separated than the other studies. However, most still fell into a higher age bracket (mean = 30) and education level was not recorded. Limited diversity within and across samples does limit the generalisability of findings to the wider population. This is a common issue across many self‐selecting health and breastfeeding support studies, which often underrecruit participants from ethnic minority backgrounds.

Women from ethnic minority backgrounds are less likely to join breastfeeding peer support groups than White women (McAndrew et al., 2012), and this may also apply to Facebook groups (La Leche League, 2020). Those who seek support online struggle to find local groups that reflect and share their experiences, with Black British mothers reporting joining American BSF groups solely for Black women to feel part of a relatable breastfeeding community (CIBII, 2018). Greater reliance on friends and family, and fewer representative online communities may also have seen COVID‐related impacts on breastfeeding support being greater for some women (Brown, 2017).

## 5 CONCLUSIONS

There is a paucity of UK research, a gap that needs to be addressed to determine the specific impact of SM group use on the UK breastfeeding context. However, this review finds that women across the countries included finding SM support beneficial. It identified that the women who seek and engage with self‐directed SM support most often are those with high levels of intention and motivation and that they perceive access to peer and professional support within virtual communities as extending their breastfeeding goals and achieved duration. Currently, many of these women stop breastfeeding before they are ready to do so. The results of this review confirm the importance of further research to understand how health professionals and wider services can draw on the benefits of SM group provision to better support women and to underpin greater investment.

## AUTHOR CONTRIBUTIONS

Holly Morse was responsible for study conception, selecting and reviewing all articles, draft manuscript completion and critical revisions. Amy Brown was responsible for draft manuscript support and critical revisions.

## CONFLICT OF INTEREST

The authors declare no conflict of interest.

## Data Availability

Data sharing is not applicable to this article as no new data were created or analysed in this study.

## APPENDIX A SUMMARY OF REVIEWED ARTICLES

**Table:**

Authors/year	Research aim	SM type/s	Sample	Method	Key findings
Alianmoghaddam et al. (2018)	Explore the quality of BF support through SM.	Internet and SM inc FB and MB	30 mothers breastfeeding babies 0–6 months (New Zealand)	Face‐to‐face interviews monthly until BF cessation or 6 months.	Mothers need reliable online information; Apps can be good for promoting BF; Information is accessed through weak ties on FB. SM should be used to support EBF.
Black et al. (2020)	Explore experiences of SM for BF and any extended BF success. Use SCT as a theoretical lens.	FB	8 women from one FB group (Ireland)	Semistructured interviews.	Increased self‐efficacy and empowerment by the online community through emotional support and information. SM potential for improved BF outcomes.
Bridges et al. (2018)	Explore experiences of BF mothers using ABA peer support FB groups and commonly discussed topics.	FB	778 wall posts with a total of 2998 comments (Australia)	Online ethnography of posts made in 15 ABA FB groups over 4 weeks in 2013.	Informational (learning) and emotional support (coping strategies) are provided by peer supporters and other mothers. Group admin also plays a vital role in overseeing discussion.
Skelton et al. (2018)	Use mothers’ attitudes and behaviours in relation to SM use to understand the effects on BF outcomes.	FB	21 women (focus group) and 12 mothers (interviews) from one FB group (US)	Online focus groups and interviews. Thematic analysis.	SM positively influences BF attitudes, knowledge, behaviours and longer BF duration.
Skelton et al. (2020)	To explore how the use of a FB BF group influences BF‐related knowledge, attitude and behaviours, through the theoretical lens of a CoP.	FB	21 women (focus group) and 12 mothers (interviews) from one FB group (US)	Online focus group and individual interviews (*n* = 12). Content analysis.	Concept of online CoP: sense of community, trust, interaction and BF promotion. Captures and stores knowledge for access. Motivated to share in group.
Robinson et al. (2019)	Examine sources of BF support in SM groups, compared with offline sources. Explore mechanism for translation into behaviour change.	FB	277 African‐American mothers from 9 FB groups (US)	Online survey analysed statistically.	FB groups provided the most support, compensating for lack elsewhere. Level of FB support correlated with the intended BF duration.
Bridges (2016)	How mothers find and share info on BF FB groups.	FB	3 FB groups were observed, followed by 23 group participants interviewed (Australia)	Case study interviews and online focus groups.	SM provides immediate, complementary support to BF mothers. Moderated forums provide trusted info.
Regan and Brown (2019)	Explore positive and negative experiences of online BF support and motivations.	FB	14 mothers breastfeeding child up to 3 years (UK)	Semistructured interviews.	Mothers were drawn to online support because it fills a gap. Support is reassuring, empathetic and convenient, but negatives include judgement, polarised debate and lack of regulation.
Lebron et al. (2020)	Examine the Babycenter BF support forum to understand the information‐seeking and information‐sharing practices of its users.	MB (Babycenter)	258 posts and 1445 corresponding comments (US)	Content analysis.	Popular topics challenges, supply, and so on. Used interview‐style questions and built consensus through agreement. Shared knowledge and encouragement—important future resource and intervention.
Wagg et al. (2019)	To document and describe the posts made within an online breastfeeding support group.	FB	501 posts and associated comments. Most from mothers with babies 6wks‐6mths. (UK)	Systematic message content analysis.	FB group used to request and receive a range of support around the clock. Creates a community that shares and celebrates.
Robinson et al. (2019)	Describe experiences of African‐American mothers using FB for BF support and their beliefs, practices and outcomes.	FB	22 Black mothers (US)	Four online focus groups.	Improved confidence for public BF and prolonged initial goals. Valued convenient access and online community. Positively influences norms.
Herron et al. (2015)	To conceptualise online BF support.	MB (Netmums)	1230 online messages, online interviews with 12 women (Ireland)	Concept analysis of messages and individual online interviews.	Support and debate with experienced mothers which is accessible, responsive and sustained by indirect reciprocity. Woman‐generated, authentic support opens doors for investment.
Wilson (2020)	Explore variables leading to sustained BF for SM group users.	FB	241 women from 17 FB BF groups 1230) (US)	Longitudinal, mixed methods repeated‐measures surveys including Perception scales and the breastfeeding confidence, knowledge and attitudes measure.	FB groups improve confidence, knowledge, attitudes and the potential for exclusive breastfeeding to 6 months. Significant results on outcomes.

Abbreviations: ABA, Australian Breastfeeding Association; BF, breastfeeding; CoP, community of practice; EBF, exclusive breastfeeding; FB, Facebook; MB, message boards; SCT, social cognitive theory; SM, social media UGC, user‐generated content.

*Source*: Data extraction form (adapted from Aveyard et al., 2016).

## APPENDIX B QUALITY APPRAISAL SUMMARY OF INCLUDED QUALITATIVE STUDIES (CRITICAL APPRAISAL SKILLS PROGRAMME UK, 2018)

**Table:**

	Met the criteria? (yes [Y], cannot tell [?], no [?])							
CASP criteria	Alianmoghaddam et al. (2018)	Black et al. (2020)	Bridges et al. (2018)	Skelton et al. (2018)	Robinson et al. (2019)	Bridges (2016)	Regan and Brown (2019)	Herron et al. (2015)
Q1: Was there a clear statement of the aims of the research?	Y	Y	Y	Y	Y	Y	Y	Y
Q2: Is a qualitative methodology appropriate?	Y	Y	Y	Y	Y	Y	Y	Y
Q3: Was the research design appropriate to the aims of the research?	Y	?	Y	Y	Y	Y	Y	Y
Q4: Was the recruitment strategy appropriate to the aims of the research?	Y	Y	Y	Y	Y	Y	Y	Y
Q5: Were the data collected in a way that addressed the research issue?	Y	Y	Y	Y	Y	Y	Y	Y
Q6: Has the relationship between researcher and participants been adequately addressed?	N	N	N	N	Y	Y	Y	Y
Q7: Have ethical issues been taken into consideration?	Y	Y	Y	Y	Y	Y	Y	Y
Q8: Was the data analysis sufficiently rigorous?	Y	Y	Y	Y	Y	Y	Y	Y
Q9: Is there a clear statement of findings?	Y	Y	Y	Y	Y	Y	Y	Y

## APPENDIX C QUALITY ASSESSMENT WITH DIVERSE STUDIES (QUADS) APPRAISAL OF INCLUDED MIXED METHODS STUDIES (HARRISON ET AL., 2021)

**Table:**

	No mention (0) General (1) Specific (2) Explicit (3)				
QuADS criteria	Wilson (2020)	Skelton et al. (2020)	Lebron et al. (2020)	Wagg et al. (2019)	Robinson et al. (2019)
Q1: Theoretical or conceptual underpinning to the research	3	2	1	2	3
Q2: Statement of research aim/s	3	3	2	3	3
Q3: Clear description of research setting and target population	3	3	2	3	3
Q4: The study design is appropriate to address the stated research aim/s	2	3	2	3	3
Q5: Appropriate sampling to address the research aim/s	1	2	1	3	3
Q6: Rationale for choice of data collection tools	2	3	2	2	2
Q7: The format and content of data collection tool is appropriate to address aims	3	3	2	2	2
Q8: Description of data collection procedure	3	3	3	3	3
Q9: Recruitment data provided	3	3	1	2	3
Q10: Justification for analytic method selected	3	3	2	3	3
Q11: The method of analysis was appropriate to answer research aim/s	2	3	2	3	3
Q12: Evidence that the research stakeholders have been considered in research design or conduct	0	1	0	0	3
Q13: Strengths and limitations critically discussed	3	3	3	3	3
